# Mulberry twig alkaloids for type 2 diabetes mellitus: a systematic review and meta-analysis

**DOI:** 10.3389/fphar.2025.1475080

**Published:** 2025-02-27

**Authors:** Guoming Chen, Ruifeng Yang, Xiaoya Yang, Jiazhen Jiang, Yudan Guo, Mengshan Xu, Yi Chen, Yining Hou, Cheng Zhang, Ning Wang, Yibin Feng

**Affiliations:** ^1^ School of Chinese Medicine, Li Ka Shing Faculty of Medicine, The University of Hong Kong, Pokfulam, Hong Kong SAR, China; ^2^ School of Traditional Chinese Medicine, Beijing University of Chinese Medicine, Beijing, China; ^3^ School of Medical Information Engineering, Guangzhou University of Chinese Medicine, Guangzhou, China; ^4^ School of First Clinical Medical College, Guangzhou University of Chinese Medicine, Guangzhou, China; ^5^ Seventh Clinical Medical College, Guangzhou University of Chinese Medicine, Guangzhou, China; ^6^ Clinical Medical College of Acupuncture-Moxibustion and Rehabilitation, Guangzhou University of Chinese Medicine, Guangzhou, China; ^7^ School of Public Health and Management, Guangzhou University of Chinese Medicine, Guangzhou, China; ^8^ School of Basic Medicine, Guangzhou University of Chinese Medicine, Guangzhou, China

**Keywords:** mulberry twig alkaloids, type 2 diabetes mellitus, systematic review, meta-analysis, traditional Chinese medicine

## Abstract

**Background:**

Diabetes has emerged as a significant global health concern, with over 95% of cases categorized as type 2 diabetes mellitus (T2DM). The disease not only imparts detrimental effects on individual health but also imposes a substantial burden on societal economics and healthcare systems. Notably, there is a paucity of meta-analyses on the efficacy of mulberry twig alkaloids (MTAs) for the treatment of T2DM.

**Methods:**

The systematic review registration number is CRD42024523218. Data were retrieved from Cochrane Library, ClinicalTrials.gov, Embase, PubMed, Ovid, Web of Science, China National Knowledge Infrastructure (CNKI), Scopus, Chongqing VIP, CINAHL, SINOMED, ChiCTR, and Wanfang Data from their inception to 1 February 2024 for herbal product-related randomized controlled trials (RCTs). The risk of bias assessment and meta-analysis were performed using ReviewManager 5.4 and STATA 15.0. TSA software version 0.9.5.10 was used to assess whether the results achieved the required information size (RIS). GRADEprofiler 3.6 software was used to estimate the quality of evidence for the outcomes.

**Results:**

Nine studies were included for a total of 10 trials with 1,178 patients. The results indicated that MTAs were more effective than placebo in reducing HbA1cglycated hemoglobin (HbA1c), and MTAs combined with hypoglycemic drugs were more effective than hypoglycemic drugs alone in reducing HbA1c, fasting blood glucose (FBG), and 2-hour postprandial glucose (2hPG). In terms of lipid control, MTAs combined with hypoglycemic drugs showed better control of triglycerides (TGs) and low-density lipoprotein (LDL) efficacy than hypoglycemic drugs alone. After MTA treatment, there was no damage to liver function compared to placebo. The safety of MTAs, whether alone or in combination with hypoglycemic drugs, was comparable to that of hypoglycemic drugs alone.

**Conclusion:**

In T2DM patients, MTAs were more effective than placebo. MTAs combined with hypoglycemic drugs showed better results than hypoglycemic drugs alone. The safety of MTAs was equivalent to that of hypoglycemic drugs. However, due to heterogeneity and possible bias, the results should still be interpreted with caution.

**Systematic Review Registration:**

https://www.crd.york.ac.uk/PROSPERO/display_record.php?RecordID=523218

## 1 Introduction

Nine out of ten patients with diabetes have type 2 diabetes mellitus (T2DM), which exhibits features more commonly observed in adults and is not associated with insulin dependence (Khan et al., 2020). Diabetes, one of the chronic metabolic diseases that are detrimental to human health, is characterized by high blood glucose levels (Zheng et al., 2018; Tinajero and Malik, 2021). Older individuals are more likely to develop T2DM, particularly if they are overweight and inactive. Epidemiological surveys indicate that with the anticipated increase in the diagnosis rate and prevalence of T2DM, approximately 10 % of the global population will be affected by T2DM by 2040 (Sun et al., 2022). In the context of insulin resistance, T2DM is caused by a progressive reduction in β-cell insulin secretion. Meanwhile, relatively inadequate insulin cannot maintain stable and healthy blood glucose levels (American Diabetes Association, 2022). In essence, the high prevalence and intricate pathological processes of the disease present significant obstacles to clinical treatment and social advancement.

Mulberry twig alkaloid (MTA) tablets are extracted from Ramulus mori (*Morus indica* L.), the principal bioactive component of polyhydroxy alkaloids, using a meticulous extraction, isolation, and purification method (Zhang et al., 2024). MTAs primarily encompass 1-deoxynojirimycin, fagopyrin, and 1,4-dideoxy-1,4-imino-d-arabinitol (Cao et al., 2021). MTAs were initially discovered in China and represent promising natural therapeutic agents for managing T2DM. Notably, MTAs received approval from the China National Medical Products Administration for the treatment of T2DM in 2020.

As α-glucosidase inhibitors, MTAs effectively hinder the binding of oligosaccharides to the cells of the small intestinal wall, thereby delaying glucose absorption and mitigating postprandial hyperglycemia (Jin et al., 2022). MTAs ameliorate glucose–lipid metabolism disorders and renal pathologies in diabetic individuals. They can also increase insulin secretion in response to glucose stimulation, significantly reduce intestinal lipid absorption, and attenuate inflammatory response (An et al., 2023). In addition to lowering postprandial blood glucose by inhibiting α-glycosidase, MTAs possess various pharmacological effects, such as improving the function of the intestine–islet axis, reducing inflammation, and regulating lipid disorders (Liu et al., 2023). Although a herbal MTA product is already on the market, a summary of additional experimental evidence would undoubtedly enhance its persuasiveness. We conducted the first systematic review and meta-analysis of MTA in the treatment of T2DM, with the aim of providing a prospective therapeutic approach for consideration in clinical medication.

## 2 Methods

### 2.1 Protocol and registration

The study adhered to the Preferred Reporting Items for Systematic Reviews and Meta-analyses (PRISMA) guidelines (Page et al., 2021). This systematic review of MTAs for T2DM was registered with the registration number CRD42024523218.

### 2.2 Inclusion criteria

The inclusion criteria were as follows: 1) population: the study subjects were adult T2DM patients according to the WHO 1999 diagnostic criteria (Alberti and Zimmet, 1998; Chinese Diabetes Society, 2018), with a glycated hemoglobin (HbA1c) level of 7%–10%. 2) Intervention: the treatment group received mulberry twig alkaloids alone or in combination with the same dose of hypoglycemic drugs. 3) Comparison: placebo or hypoglycemic drugs, with no restrictions on the dosage forms (oral or injection). 4) Outcome: glycemic control, lipid control, and safety outcomes, such as liver function, kidney function, and total adverse event rate. 5) Study type: RCTs with no restrictions on language.

### 2.3 Exclusion criteria

The exclusion criteria were as follows: 1) duplicate literature; 2) animal or cell experiments; 3) subjects with other types of diabetes or severe complications; 4) interventions involving other therapies; and 5) the full text could not be obtained, or the data of the article were insufficient for analysis.

### 2.4 Search strategy

Data were retrieved from 11 databases, namely, Web of Science, China National Knowledge Infrastructure (CNKI), Cochrane Library, Scopus, PubMed, Ovid, Embase, CINAHL, SINOMED, Wanfang Data, and Chongqing VIP, and two registries, ClinicalTrials.gov and ChiCTR, from the inception of the databases to 1 February 2024 to find eligible RCTs, and the basic search formula was (Type 2 Diabetes Mellitus [Title/Abstract]) AND (mulberry twig alkaloids [Title/Abstract]) AND [(Randomized controlled trial [Title/Abstract]). Different search formulas were constructed according to the characteristics of each database (Supplementary Table S1).

### 2.5 Study screening and data extraction

The acquired bibliography obtained by searching the databases according to the retrieval strategy was imported into EndNote 20 software. By referring to the steps of the PRISMA flow diagram, the literature was screened step by step by selecting the content, then scanning the title and abstract, and finally reading the full version. The following information was extracted: 1) first author and publication date; 2) basic line characteristics of the patients, such as sample size, age, and disease duration; 3) intervention and treatment courses in the MTA group and the placebo or hypoglycemic drug group; and 4) glycemic control, lipid control, body weight change, and safety outcomes.

### 2.6 Risk of bias assessment

The Cochrane risk of bias assessment tool in RevMan 5.4 software was chosen to assess the risk of bias in the included studies (Sterne et al., 2019). Three assessments, namely, low risk, high risk, or uncertain bias were determined according to the evaluation of seven items in the included literature. The above assessments were completed by two researchers independently (RY and XY). If there was any disagreement, this was discussed or decided by a third researcher (GC).

### 2.7 Statistical analysis

RevMan 5.4 software was used for meta-analysis of the change value of the outcome before and after treatment (Wan et al., 2014; Luo et al., 2018). When *I* ^*2*^ < 50%, we use the fixed effects model, whereas when *I* ^*2*^ ≥ 50%, we switched to the random-effects model. The continuous variable and its 95% confidence interval (CI) were described as standardized mean difference (SMD), and the relative risk (RR) was conveyed as the outcome of the dichotomous variable. STATA 15.0 software was used to perform sensitivity analysis and to detect publication bias. TSA software version 0.9.5.10 was used to estimate whether the improvement in the depression symptom index and blood glycemic control index achieved the required information size (RIS). The quality of evidence was evaluated using GRADEprofiler 3.6 software (GRADE, Grading of Recommendations Assessment, Development and Evaluation) and classified into four levels, namely, high, moderate, low, and very low.

## 3 Results

### 3.1 Selection and identification of studies

**FIGURE 1:**
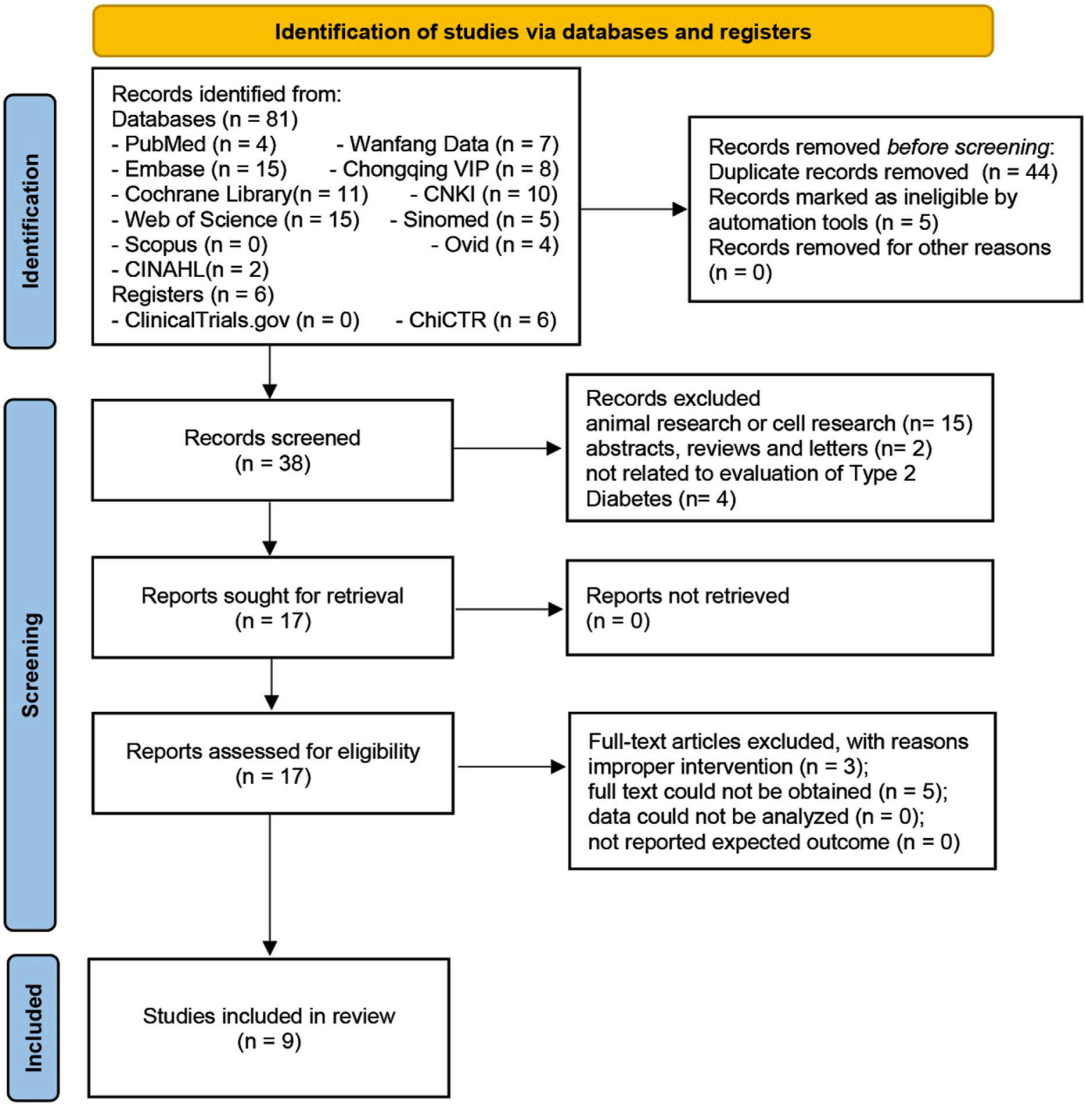
Flow chart of literature screening.

Eighty-seven studies were initially found in eleven databases and two registers, including fifteen in Web of Science, four in PubMed, four in Ovid, fifteen in Embase, eleven in Cochrane Library, two in CINAHL, seven in Wanfang Data, ten in CNKI, eight in Chongqing VIP, five in SINOMED, two in ClinicalTrials.gov, and six in ChiCTR, while the Scopus database did not find the relevant literature. Forty-four duplicate articles were excluded, and thirty-four articles were removed during primary and secondary screenings. Finally, nine articles involving 10 trials (Chen, 2016; Li et al., 2016; Qu et al., 2021; Huang et al., 2022; Qu et al., 2022; Meng et al., 2023; Yang and Ning, 2023; Yuan F. et al., 2023; Yuan L. et al., 2023) were included for meta-analysis (Figure 1).

### 3.2 Basic information of the included studies

All the included studies were conducted in China. A total of 1,178 patients, including 647 patients in the MTA group and 531 in the placebo or hypoglycemic drug group, were finally included in the analysis, and their baseline characteristics, including age, duration, and HbA1c level, were generally consistent (Table 1).

**TABLE 1:** Basic data of the included studies.

Included study	Male subjects/female subjects (No.)		Age/year		Disease duration		HbA1c/%		Treatment Course/week
	T	C	T	C	T	C	T	C	
Chen (2016)	10/6	8/6	52.56 ± 4.03	52.29 ± 3.27	NR	NR	7.48 ± 0.38	7.52 ± 0.41	18
Qu et al. (2022)	41/36	42/32	54.6 ± 8.9	55.2 ± 9.0	13 (7–36) m	10.5 (5–24) m	7.7 ± 0.8	7.6 ± 0.8	12
Qu et al. (2021)	156/165	118/104	54.9 ± 9.41	54.2 ± 9.01	24 (6–60) m	24 (8–60) m	7.90 ± 0.79	7.95 ± 0.87	24
Li et al. (2016)	8/15	5/10	56 ± 9.71	57 ± 6.70	36 ± 6, 18 m	30 ± 9, 12 m	8.30 ± 0.91	8.24 ± 0.95	24
Yang and Ning, 2023 (A)	40	19	57.41 ± 4.53	57.77 ± 4.66	3.00 ± 1.01 y	3.17 ± 0.98 y	7∼10	7∼10	24
Yuan et al. (2023a)	21/21	22/21	66.85 ± 6.02	67.84 ± 4.92	3.81 ± 0.69 y	3.64 ± 0.77 y	NR	NR	8
Yang and Ning, 2023 (B)	41	19	58.13 ± 3.66	57.77 ± 4.66	3.20 ± 0.94 y	3.17 ± 0.98 y	7∼10	7∼10	24
Huang et al. (2022)	22/13	21/14	59.32 ± 11.57	59.48 ± 11.42	4.45 ± 1.32 y	4.37 ± 1.28 y	7.96 ± 1.47	7.92 ± 1.49	12
Yuan et al. (2023b)	23/19	26/16	50.92 ± 7.80	50.54 ± 7.94	5.10 ± 1.58 y	5.26 ± 1.74 y	8.94 ± 2.11	8.80 ± 2.24	24
Meng et al. (2023)	8/2	5/5	48.40 ± 13.76	51.40 ± 9.06	8.60 ± 4.99 y	7.80 ± 5.12 y	9.65 (8.68, 9.80)	9.70 (9.38, 9.80)	12

Note: T, treatment group; C, control group; mean ± SD; median (Q1 and Q3); NR, not reported.

The hypoglycemic drugs in the control group included insulin, metformin, acarbose, sitagliptin, and dapagliflozin. Two studies (Chen, 2016; Qu et al., 2022) used MTAs versus placebo. Three studies (Li et al., 2016; Qu et al., 2021; Yang and Ning, 2023) adopted MTAs versus hypoglycemic drugs. Five studies (Huang et al., 2022; Meng et al., 2023; Yang and Ning, 2023; Yuan F. et al., 2023; Yuan L. et al., 2023) applied MTAs in combination with hypoglycemic drugs and compared the results with those obtained from hypoglycemic drugs alone. Glycemic control outcomes included glycated hemoglobin (HbA1c), fasting blood glucose (FBG), and 2-h postprandial glucose (2hPG). Lipid control outcomes included total cholesterol (TC), triglycerides (TGs), low-density lipoprotein (LDL), and high-density lipoprotein (HDL). Weight change outcomes included body weight change and body mass index (BMI). Safety outcomes included total adverse event rate, alanine transaminase (ALT), aspartate aminotransferase (AST), and creatinine (Cr) (Table 2).

**TABLE 2:** Intervention and key finding of the included studies.

Included study	Intervention		Key finding
	T	C	
Chen (2016)	MTA 50–100 mg, TID	MTA placebo 50–100 mg, TID	MTAs significantly reduced HbA1c, FBG, 2hPG, TC, and TGs compared to placebo. No abnormalities in liver or kidney function were observed.
Qu et al. (2022)	MTA 50–100 mg, TID	MTA placebo 50–100 mg, TID	Changes in FBG, 1hPG, and 2hPG differed significantly between the MTA and placebo groups, but differences in body weight and BMI were not significant.
Qu et al. (2021)	MTA 50–100 mg, TID + acarbose placebo, TID	Acarbose 50–100 mg, TID + MTA placebo, TID	MTAs exhibited equivalent hypoglycemic effects to acarbose. The incidence of total adverse events and gastrointestinal disorders was lower following MTA treatment than acarbose treatment.
Li et al. (2016)	MTA 50–100 mg, TID + acarbose placebo 50 mg, TID	Acarbose 50 mg, TID + MTA placebo 50–100 mg, TID	HbA1c, 1hPG, and 2hPG levels in the MTA group significantly decreased from baseline, without any significant differences compared with the acarbose group.
Yang and Ning, 2023 (A)	MTA 50–100 mg, TID	Metformin 0.5–1 g, BID	2hPG in the MTA group was lower than that in the metformin group after treatment.
Yuan et al. (2023a)	MTA 50–100 mg, QD + metformin 0.85 g, BID	Metformin 0.85 g, BID	The MTA group exhibited reduced 2hPG and HOMA-IR levels and elevated C-peptide and HOMA-β levels compared to the metformin group. There was no significant difference in the rate of adverse events between the two groups.
Yang and Ning, 2023 (B)	MTA 50–100 mg, TID + metformin 0.5–1 g, BID	Metformin 0.5–1 g, BID	FBG, 2hPG, and HOMA-IR in the MTA + metformin group were lower than in the metformin group after treatment.
Huang et al. (2022)	MTA 50–100 mg, TID + sitagliptin 100 mg, QD	Sitagliptin 100 mg, QD	MTAs combined with sitagliptin can decrease the levels of HbA1c, FPG, TC, LDL, and TG, and increase HOMA-β compared to sitagliptin alone.
Yuan et al. (2023a)	MTA 50–100 mg, TID + dagliazine 10 mg, QD	Dagliazine 10 mg, QD	MTAs combined with dapagliflozin showed significantly lower levels of FBG, 2hPG, and HbA1c than dapagliflozin alone. No significant additional adverse reactions were observed.
Meng et al. (2023)	MTA 50–100 mg, TID + insulin 0.4–0.5 IU/kg	Insulin 0.4–0.5 IU/kg	Compared with the insulin group, the insulin + MTA group showed improved HbA1c, FBG, 1hPG, fasting blood lipid, and postprandial blood lipid indicators.

Note: T, treatment group; C, control group; MTAs, mulberry twig alkaloids; 1hPG, 1-hour postprandial glucose; 2hPG, 2-hour postprandial glucose; FBG, fasting blood glucose; HbA1c, glycated hemoglobin; LDL, low-density lipoprotein; TC, total cholesterol; TG, triglycerides; HOMA-β, homeostatic model assessment for β cells; HOMA-IR, homeostatic model assessment for insulin resistance.

### 3.3 Quality assessment

**FIGURE 2:**
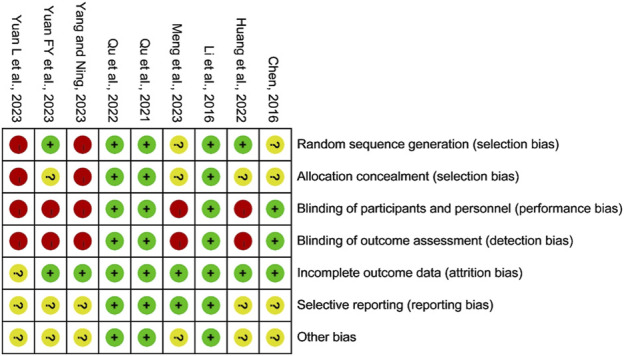
Risk of the bias graph plot.

Among the nine included studies, three articles (Li et al., 2016; Huang et al., 2022; Yuan F. et al., 2023) used the random code or random number method. Two articles (Qu et al., 2021; Qu et al., 2022) used the data analysis system for electronic data capture clinical trial central randomization system to randomly allocate and dispense drugs. Two articles (Yang and Ning, 2023; Yuan L. et al., 2023) were based on the willingness of patients, and the rest were randomized groups that did not describe the specific method. One article (Li et al., 2016) used unique identification numbers to allocate concealment. All patients and researchers were blinded in four articles (Chen, 2016; Li et al., 2016; Qu et al., 2021; Qu et al., 2022). Four articles (Chen, 2016; Li et al., 2016; Qu et al., 2021; Qu et al., 2022) reported the situation and specific reasons for shedding. One article (Yuan L. et al., 2023) did not report patient dropout, while no patients dropped out in the remaining articles. The study protocols of four articles (Li et al., 2016; Qu et al., 2021; Qu et al., 2022; Meng et al., 2023) were registered (Figure 2).

### 3.4 Meta-analysis results

#### 3.4.1 Glycemic control outcomes

Seven studies (Chen, 2016; Li et al., 2016; Qu et al., 2021; Huang et al., 2022; Qu et al., 2022; Meng et al., 2023; Yuan F. et al., 2023) reported HbA1c before and after the treatment. MTAs were more effective than placebo in reducing HbA1c (SMD = −0.85, 95% CI [−1.16, −0.55], *P* < 0.00001, *I* ^*2*^ = 0%) but showed no statistical difference compared to acarbose (SMD = −0.06, 95% CI [−0.23, 0.11], *P* = 0.48, *I* ^*2*^ = 0%). MTAs combined with hypoglycemic drugs were more effective than hypoglycemic drugs alone (SMD = −0.70, 95% CI [−1.00, −0.39], *P* < 0.00001, *I* ^*2*^ = 0%).

Eight studies (Chen, 2016; Li et al., 2016; Qu et al., 2021; Huang et al., 2022; Meng et al., 2023; Yang and Ning, 2023; Yuan F. et al., 2023; Yuan L. et al., 2023) reported FBG change. MTAs demonstrated no statistical advantage over hypoglycemic drugs in reducing FBG (SMD = −0.03, 95% CI [−0.19, 0.14], *P* = 0.74, *I* ^*2*^ = 0%). MTAs combined with hypoglycemic drugs showed greater efficacy compared to hypoglycemic drugs alone (SMD = −0.63, 95% CI [−1.04, 0.23], *P* = 0.002, *I* ^*2*^ = 65%).

**FIGURE 3:**
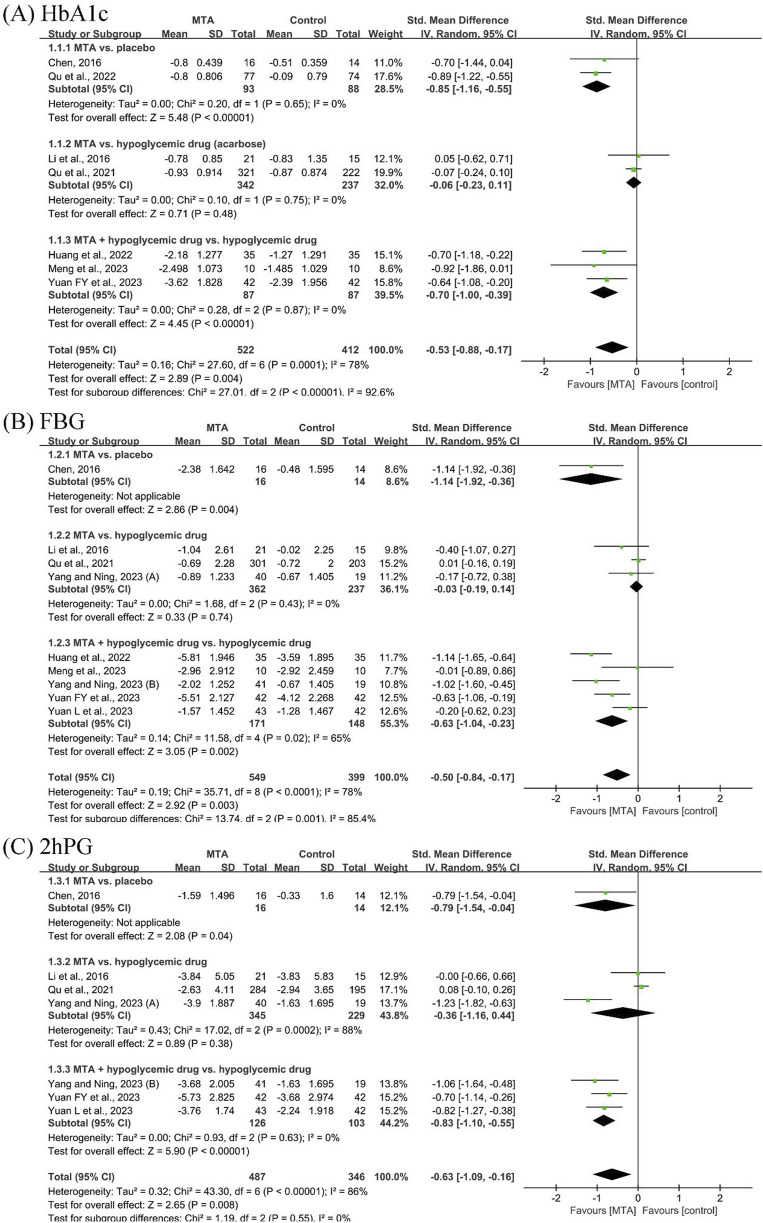
Forest plots of glycemic control outcomes: **(A)** glycated hemoglobin (HbA1c), **(B)** fasting blood glucose (FBG), and **(C)** 2-hour postprandial glucose (2hPG).

Six studies (Chen, 2016; Li et al., 2016; Qu et al., 2021; Yang and Ning, 2023; Yuan F. et al., 2023; Yuan L. et al., 2023) reported 2hPG results. The effects of MTAs and hypoglycemic drugs were found to be indistinguishable (SMD = −0.36, 95% CI [−1.16, 0.44], *P* = 0.38, *I* ^*2*^ = 88%). The MTA group demonstrated superior efficacy compared to hypoglycemic drugs alone (SMD = −0.83, 95% CI [−1.10, −0.55], *P* < 0.00001, *I* ^*2*^ = 0%) (Figure 3).

#### 3.4.2 Lipid control outcomes

**FIGURE 4:**
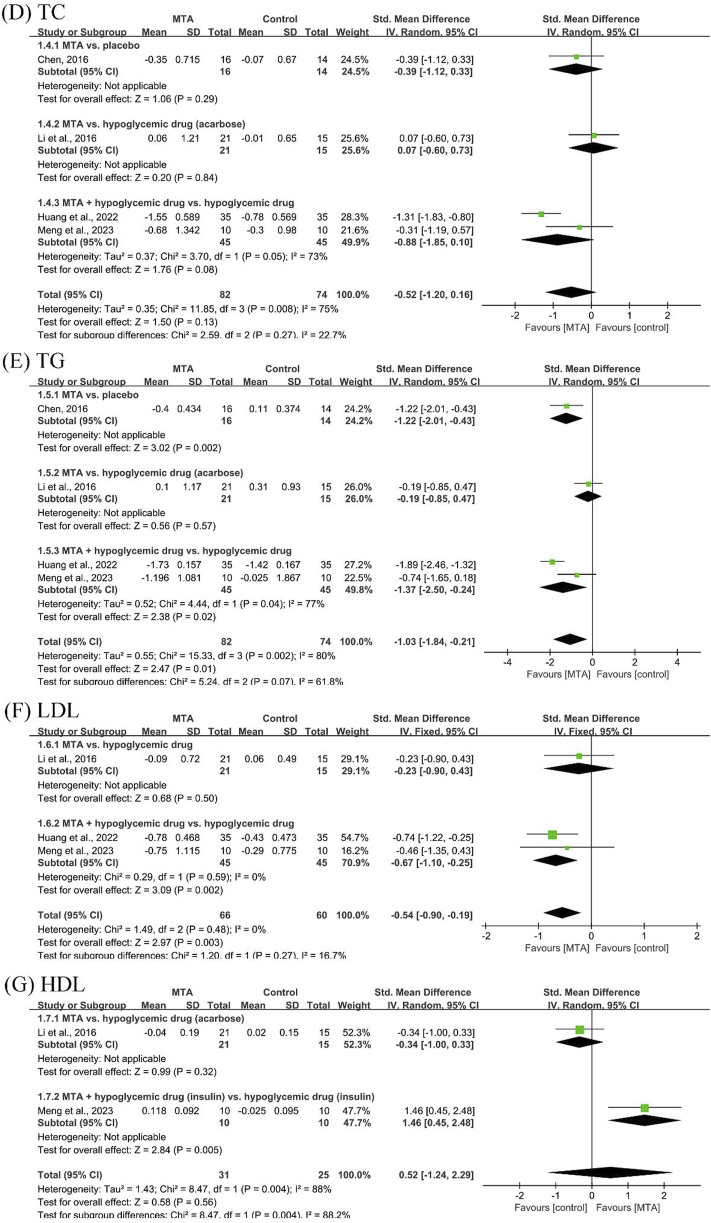
Forest plots of lipid control outcomes: **(D)** total cholesterol (TC), **(E)** triglycerides (TG), **(F)** low-density lipoprotein (LDL), and **(G)** high-density lipoprotein (HDL).

Four studies (Chen, 2016; Li et al., 2016; Huang et al., 2022; Meng et al., 2023) reported lipid control outcomes, including TC and TG. The results showed that further studies are needed to demonstrate the efficacy of MTAs in reducing TC and increasing HDL. MTAs in combination with hypoglycemic drugs showed better control of TG (SMD = −1.37, 95% CI [−2.50, −0.24], *P* = 0.02, *I* ^*2*^ = 77%) and LDL (SMD = −0.67, 95% CI [−1.10, −0.25], *P* = 0.002, *I* ^*2*^ = 0%) efficacy compared to hypoglycemic drugs alone (Figure 4).

#### 3.4.3 Body weight outcomes

**FIGURE 5:**
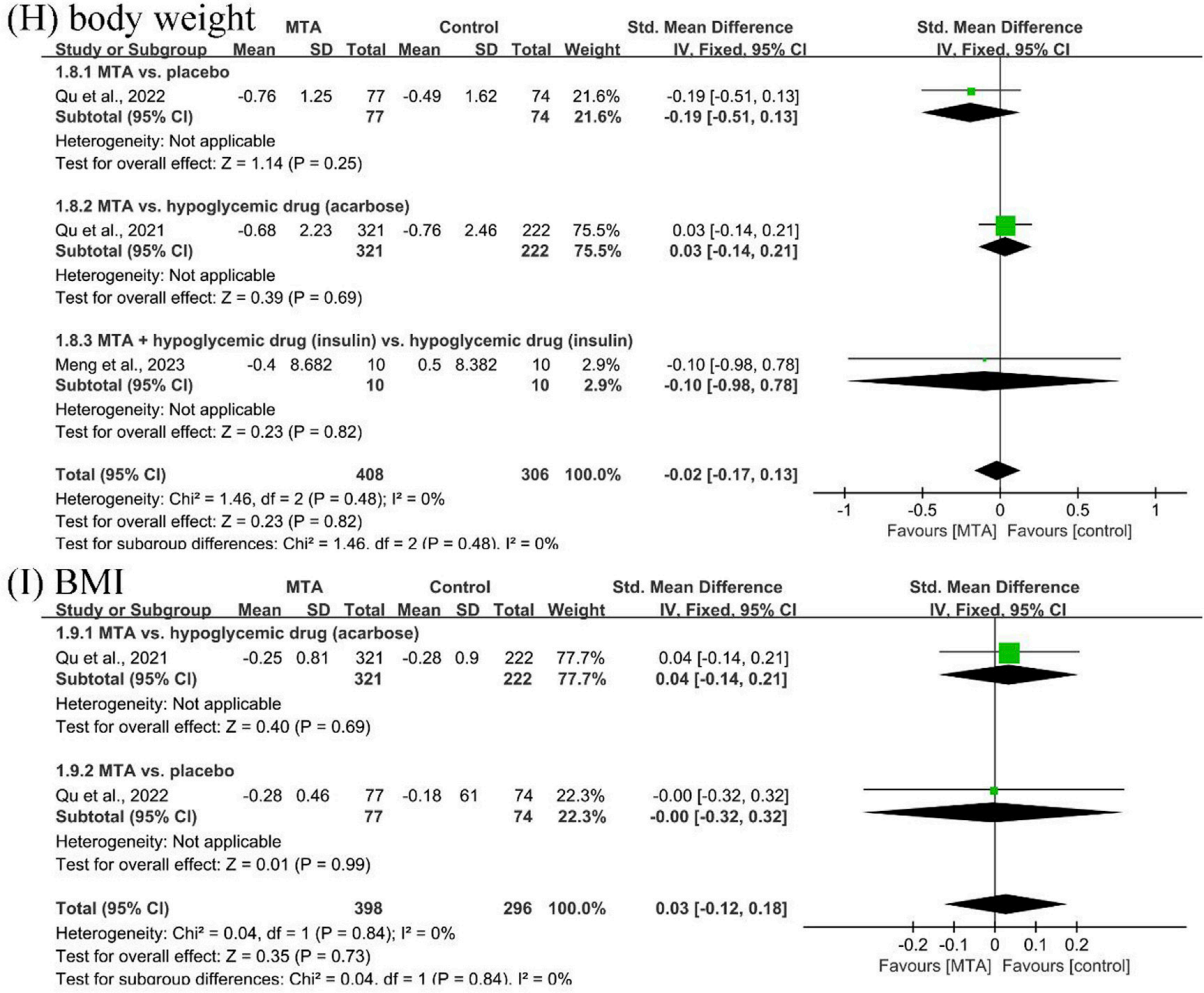
Forest plot of **(H)** body weight change and **(I)** body mass index (BMI).

Three studies (Qu et al., 2021; Qu et al., 2022; Meng et al., 2023) reported body weight change, and two of them reported the BMI outcome (Qu et al., 2021; Qu et al., 2022). The results showed that MTAs had no potential to reduce body weight (SMD = −0.02, 95% CI [−0.17, 0.13], *P* = 0.82, *I* ^*2*^ = 0%) or BMI (SMD = 0.03, 95% CI [−0.02, 0.18], *P* = 0.73, *I* ^*2*^ = 0%) (Figure 5).

#### 3.4.4 Safety outcomes

Eight studies (Chen, 2016; Li et al., 2016; Qu et al., 2021; Huang et al., 2022; Qu et al., 2022; Meng et al., 2023; Yuan F. et al., 2023; Yuan L. et al., 2023) documented the incidence and number of adverse events. One study (Chen, 2016) specifically mentioned that no serious adverse events were detected in either the placebo or MTA groups, and the reported adverse events were self-limiting and did not recur. Although the safety of MTAs versus placebo could not be confirmed, the safety of MTAs alone (RR = 0.96, 95% CI [0.84, 1.10], *P* = 0.56, *I* ^*2*^ = 0%) or in combination with hypoglycemic drugs (RR = 1.40, 95% CI [0.75, 2.62], *P* = 0.29, *I* ^*2*^ = 0%) was comparable to that of hypoglycemic drugs alone.

**FIGURE 6:**
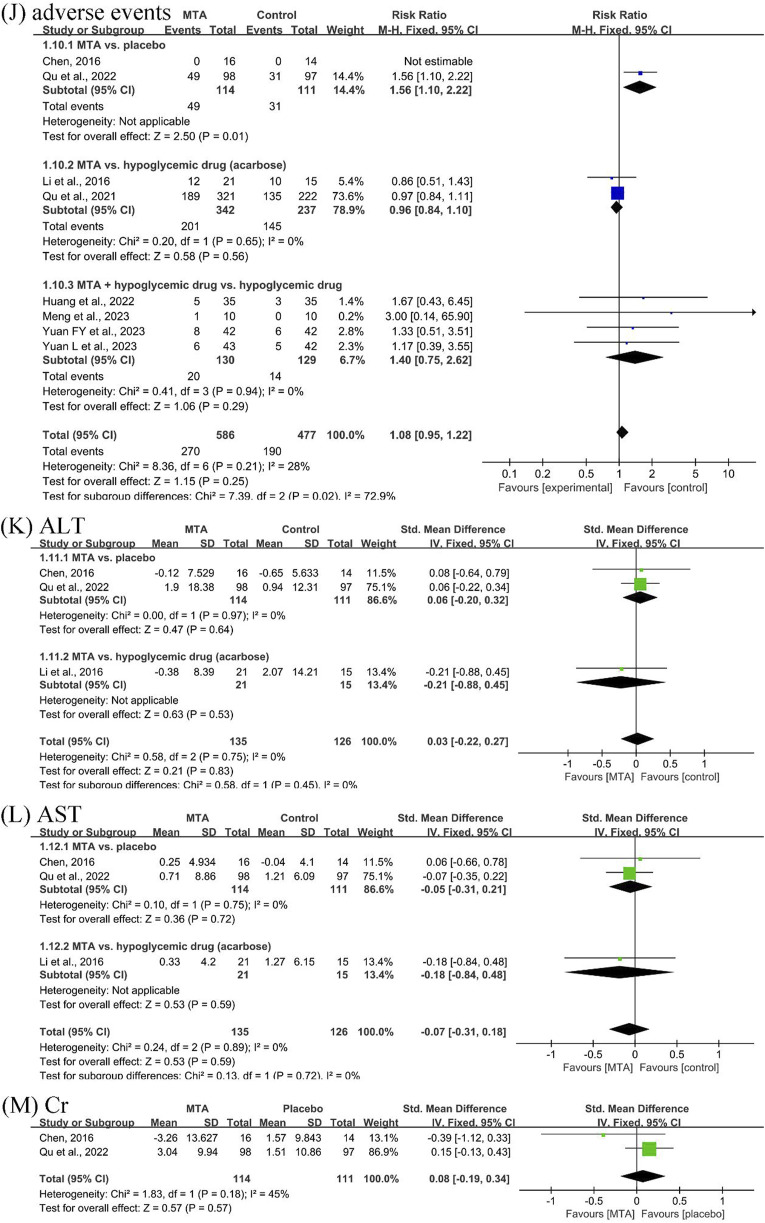
Forest plots of safety outcomes: **(J)** total adverse events, **(K)** alanine transaminase (ALT), **(L)** aspartate aminotransferase (AST), and **(M)** creatinine (Cr).

Three studies (Chen, 2016; Li et al., 2016; Qu et al., 2022) reported ALT and AST. After MTA treatment, there was no damage to liver function compared to placebo (SMD = −0.06, 95% CI [−0.20, 0.32], *P* = 0.64, *I* ^*2*^ = 0%). Additionally, two studies (Chen, 2016; Qu et al., 2022) reported the Cr outcome, which showed no effect on renal function compared to placebo after treatment (SMD = −0.08, 95% CI [−0.19, 0.34], *P* = 0.57, *I* ^*2*^ = 45%) (Figure 6).

### 3.5 Sensitivity analysis and subgroup analysis

Sensitivity analysis showed that for the 2hPG outcome in the MTA vs. hypoglycemic drug subgroup, there was no statistical change in the results after excluding one study (Yang and Ning, 2023), but the heterogeneity value *I* ^*2*^ decreased to 0% (Supplementary Figure S1). The reasons for the heterogeneity may be that the study did not use blinding methods, and the hypoglycemic drug in this study was metformin, while the other two studies used acarbose. Other meta-analysis results and heterogeneity did not significantly change after eliminating the included studies one by one. The metaninf command results suggested that the pooled effect sizes of the remaining studies were still within the 95% CI after the included studies were eliminated one by one for outcomes with high heterogeneity (Supplementary Figure S2).

Subgroup analyses of adverse events were performed according to the type of adverse event. Seven studies (Li et al., 2016; Qu et al., 2021; Huang et al., 2022; Qu et al., 2022; Meng et al., 2023; Yuan F. et al., 2023; Yuan L. et al., 2023) reported the types and numbers of adverse events. After grouping by type of adverse reaction, the incidence rate of gastrointestinal disorders (RR = 1.00, 95% CI [0.40, 2.50], *P* = 1.00, *I* ^*2*^ = 72%), edema (RR = 2.97, 95% CI [0.31, 27.95], *P* = 0.34, *I* ^*2*^ = 0%), hypoglycemia (RR = 0.56, 95% CI [0.12, 2.56], *P* = 0.45, *I* ^*2*^ = 0%), and urinary tract infection (RR = 1.42, 95% CI [0.29, 7.11], *P* = 0.67, *I* ^*2*^ = 0%) showed no discernible difference between the MTA and the control groups (Supplementary Figure S3).

### 3.6 Publication bias

Since all outcomes included fewer than 10 studies, the Egger’s test, instead of the funnel plot, was utilized to detect potential publication bias. Except for the FBG and 2hPG outcomes, the *p*-values for the remaining outcomes were greater than 0.05, suggesting no significant publication bias (Supplementary Table S2). The non-parametric trim-and-fill method was further applied to the FBG and 2hPG outcomes. There were no indications of publication bias using the trim-and-fill method (no new studies were added), and no significant change occurred before and after trimming, indicating that possible publication bias had minimal impact on the results. The power of the Egger’s test was reduced by the small number of studies. Publication bias results should be interpreted with caution (Supplementary Figure S4).

### 3.7 Trial sequential analysis and GRADE evaluation

**FIGURE 7:**
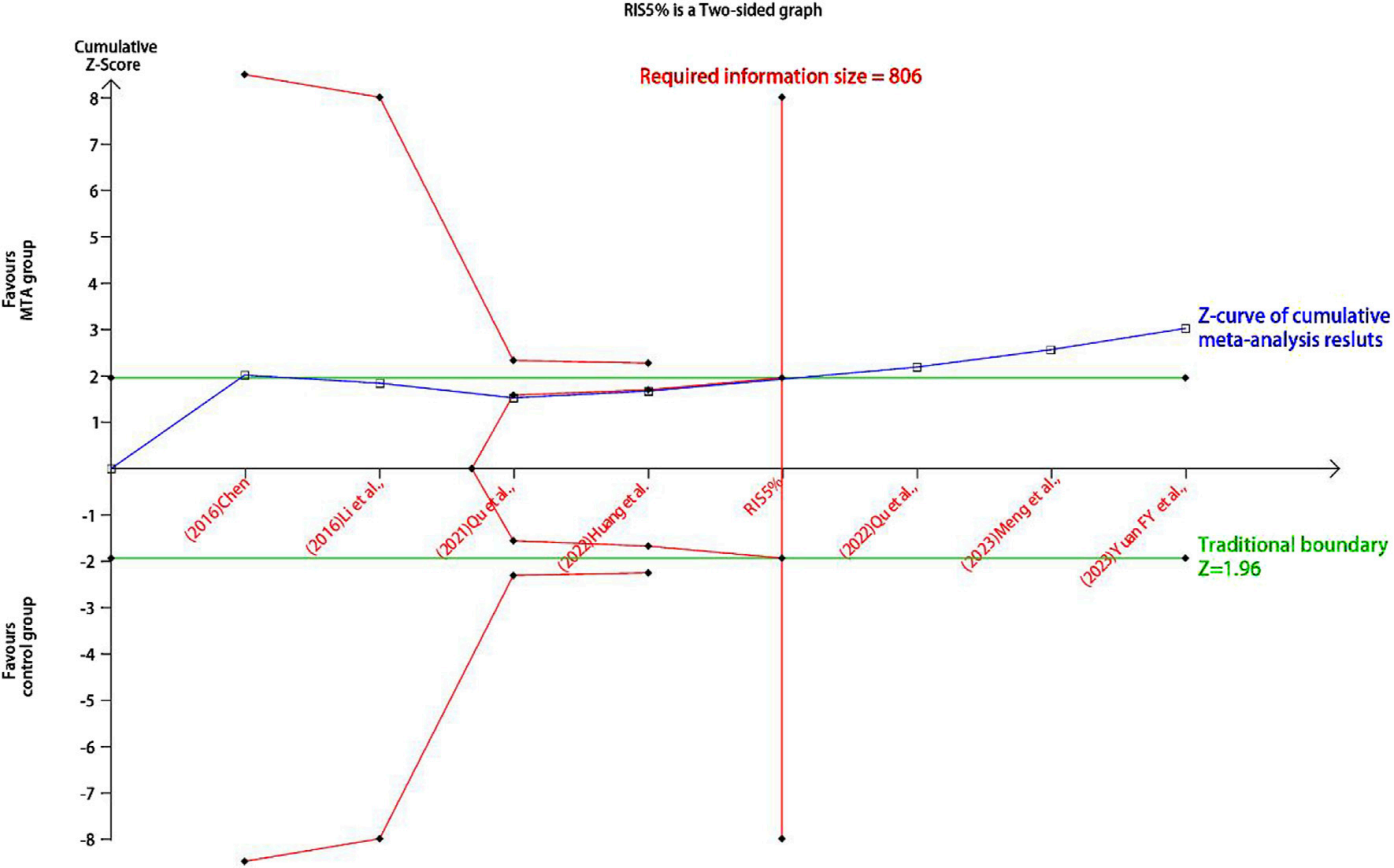
Trial sequential analysis of the HbA1c outcome.

Trial sequential analysis was conducted to identify the results for false positives. We set the type 1 error to 5%, the test power to 80%, and the RIS option to “Empirical.” The blue Z-curve for the combined effect sizes of the HbA1c, FBG, 2hPG, TG, and LDL outcomes in the MTA group versus the control group crossed the horizontal green line of the traditional threshold and the red TSA threshold. Furthermore, the outcomes reached the RIS number, which indicated that the sample size was sufficient to support conclusions (Figure 7; Supplementary Figure S5).

The quality of evidence for 13 outcomes was evaluated using the GRADEprofiler 3.6 software. The evaluation resulted in 1 moderate-quality evidence of HbA1c and 3 very low-quality evidence of FBG, 2hPG, and HDL. The evidence quality of 9 outcomes including TC, TG, LDL, body weight, BMI, total adverse events, ALT, AST, and Cr was low. The majority of the reasons for downgrading were due to indirectness (Supplementary Table S3).

## 4 Discussion

Our meta-analysis results showed that statistically significant differences between MTAs and placebo or MTAs in combination with hypoglycemic drugs for the outcome indicators of HbA1c, FBG, 2hPG, TG, and LDL. MTAs did not show obvious advantages in direct comparison with hypoglycemic drugs such as metformin and acarbose in terms of glycemic control outcomes, lipid control outcomes, or body weight loss.

MTAs, as natural compounds extracted from *Ramulus mori*, can inhibit the activity of α-glucosidase both *in vivo* and *in vitro*. Dynamic analysis and molecular docking methods revealed the selective inhibitory effects and mechanisms of MTAs and their main active ingredients on different α-glucosidases. The research results indicated that MTAs possessed selective inhibitory effects on the activity of maltase and sucrase to reduce hyperglycemia with a reversible competitive inhibition, while their inhibitory effect on α-amylase is weaker than that of acarbose (Liu et al., 2019). Furthermore, Lei et al. (2022) showed that MTAs improve the function and morphology of islet β cells, which may be related to the downregulation of the β-cell dedifferentiation marker ALDH1A3 and the upregulation of β-cell identification genes. MTAs offer benefits in the management of T2DM, such as the ability to enhance lipid metabolism and reduce blood glucose levels. These effects may be associated with increased levels of glucagon-like peptide 1, modulation of the gut microbiota, and a reduction in the degree of ileal and systemic inflammation (Liu et al., 2021). The finding of Li et al. (2023) indicated that MTAs can improve diabetic nephropathy in diabetic rat models by reducing renal inflammation and renal fibrosis. The main component of MTAs, 1-deoxynojirimycin enhanced its insulin-resistance-improving effect by inhibiting the activity of the toll-like receptor 4/nuclear factor-κB (TLR4/NF-κB) signaling pathway and the expression of the suppressor of cytokine signaling 3 (SOCS3) and increasing the expression of tight junction proteins and phosphorylated IRS1 (Tyr896) to total IRS1 (Ren et al., 2022). TLR4 is an extracellular receptor that detects a broad spectrum of pathogens and damage-associated molecular patterns. NF-κB proteins are a group of transcription factors that play a crucial role in the regulation of inflammation and immune responses. With its minimal side effects and therapeutic impact in the treatment of T2DM, MTAs offer a wide range of applications in clinical practice.

Furthermore, using MTA did not place an extra burden on liver and kidney function. A relevant study has shown that MTAs can alleviate the clinical symptoms of pre-diabetic patients by improving insulin sensitivity, reducing serum nesfatin-1 levels, and increasing serum glucose transporter 4 (GLUT4) levels (Wang et al., 2023). In addition, the daily cost of MTAs is approximately 1.5–3¥. Because of its relativelylow cost and high safety, it has the potential to provide a new option for the treatment of T2DM when combined with existing hypoglycemic drugs.

This study was the first systematic review of MTAs for T2DM; we analyzed glycemic control outcomes, lipid control outcomes, body weight loss, and safety outcomes to evaluate their effects. We hope that more research on MTAs for T2DM will follow, and it would be valuable to conduct studies comparing hypoglycemic drugs with placebos, thus discovering and developing a safe complementary therapy for T2DM.

## 5 Limitations

The study presented several limitations. First, all participants were Chinese, which hinders the ability to make cross-regional or cross-ethnic efficacy comparisons. Second, the limited number of studies that met the inclusion criteria and the small sample sizes within each intervention subgroup affected the precision of the results. Third, the majority of the analyzed studies administered MTAs at a dosage of 50 mg/day for 4 weeks, followed by 100 mg/day, but failed to investigate the dose–response relationship of MTAs to determine their optimal dosage. Finally, the duration of treatment observation ranged from 2 to 6 months, with a lack of long-term follow-up data. It is important to consider the heterogeneity of the outcomes, which may be attributed to various factors such as the quality of the literature, the diversity of interventions, the number of doses, and the length of the treatment.

## 6 Conclusion

MTAs demonstrated a significant effect on improving glycemic control indicators, such as HbA1c, FBG, and 2hPG, in addition to better control of TG levels. In terms of safety outcomes, such as total adverse events, ALT, AST, and Cr, MTAs showed acceptable safety. However, more evidence is needed to determine its effects on glycemic control, TG reduction, and weight loss, especially in direct comparisons with hypoglycemic drugs such as metformin or acarbose. However, due to heterogeneity and the limited sample size, the results should be interpreted with caution.

## Data Availability

The original contributions presented in the study are included in the article/Supplementary Material; further inquiries can be directed to the corresponding author.

## Abbreviations

2hPG, 2-hour postprandial glucose; ALT, alanine transaminase; AST, aspartate aminotransferase; Cr, creatinine; FBG, fasting blood glucose; HbA1c, glycated hemoglobin; HDL, high-density lipoprotein; LDL, low-density lipoprotein; MTAs, mulberry twig alkaloids; RCT, randomized controlled trial; RIS, required information size; TC, total cholesterol; TGs, triglycerides; T2DM, type 2 diabetes mellitus.
