# Supplementary material for: Mulberry twig alkaloids for type 2 diabetes mellitus: a systematic review and meta-analysis
Source: Front Pharmacol. 2025 Feb 27;16:1475080. doi: 10.3389/fphar.2025.1475080 (PMC11903429; doi:10.3389/fphar.2025.1475080)
Supplement: Supplementary file 1 [file DataSheet1.docx]

**Supplementary materials**

**Supplementary Table S1.** Retrieval strategy table.

**Supplementary Figure S1.** Sensitivity analysis of 2hPG outcome after excluded 1 study (Yang & Ning, 2023).

**Supplementary Figure S2.** Sensitivity analysis plots.

**Supplementary Figure S3.** Subgroup analysis of adverse events according to type.

**Supplementary Figure S4**. Publication bias plots.

**Supplementary Table S2**. Egger test of outcomes.

**Supplementary Figure S5**. Trial sequential analysis of FBG, 2hPG, TG, and LDL outcomes.

**Supplementary Table S3**. GRADE evaluation of evidence quality.

**Supplementary Table S4.** PRISMA 2020 checklist.

**Supplementary Table S1.** Retrieval strategy table.

PubMed

#1 Mulberry Twig Alkaloids[Mesh]

#2 Diabetes Mellitus, Type 2[Mesh]

#3 ((((Mulberry Twig Alkaloids Tablets[Title/Abstract]) OR (Mulberry Twig Alkaloids[Title/Abstract])) OR (Mulberry Twig[Title/Abstract])) OR (Ramulus Mori[Title/Abstract])) OR (Sangzhi[Title/Abstract])

#4 ((((((((((NIDDM[Title/Abstract]) OR (MODY[Title/Abstract])) OR (Ketosis Resistant Diabetes Mellitus[Title/Abstract])) OR (Maturity Onset Diabetes Mellitus[Title/Abstract])) OR (Non Insulin Dependent Diabetes Mellitus[Title/Abstract])) OR (Stable Diabetes Mellitus[Title/Abstract])) OR (Type II Diabetes Mellitus[Title/Abstract])) OR (Slow Onset Diabetes Mellitus[Title/Abstract])) OR (Type 2 Diabetes Mellitus[Title/Abstract])) OR (Type 2 Diabetes[Title/Abstract])) OR (Adult Onset Diabetes Mellitus[Title/Abstract])

#5 (clinical[tiab] AND trial[tiab]) OR "clinical trials as topic"[mesh] OR "clinical trial"[pt] OR random*[tiab] OR "random allocation"[mesh] OR "therapeutic use"[sh]

#6 #3 AND #4 AND #5

Web of Science

#1 ((((TS=(Mulberry Twig Alkaloids Tablets)) OR TS=(Mulberry Twig Alkaloids)) OR TS=(Mulberry Twig)) OR TS=(Ramulus Mori)) OR TS=(Sangzhi)

#2 ((((((((((TS=(NIDDM)) OR TS=(MODY)) OR TS=(Ketosis Resistant Diabetes Mellitus)) OR TS=(Maturity Onset Diabetes Mellitus)) OR TS=(Non Insulin Dependent Diabetes Mellitus)) OR TS=(Stable Diabetes Mellitus)) OR TS=(Adult Onset Diabetes mellitusa)) OR TS=(Type II Diabetes Mellitus)) OR TS=(Slow Onset Diabetes Mellitus)) OR TS=(Type 2 Diabetes Mellitus)) OR TS=(Type 2 Diabetes)

#3 (((((TS=(randomized controlled trial)) OR TS=(random controlled trial)) OR TS=(clinical trial)) OR TS=(randomized)) OR TS=(random)) OR TS=(clinical)

#4 #1 AND #2 AND #3

Embase

#8. #5 AND #6 AND #7

#7. #3 OR #4

#6. #1 OR #2

#5. 'clinical':ti,ab AND 'trial':ti,ab OR 'clinical trial'/exp OR random* OR 'drug therapy':lnk

#4. 'niddm':ti,ab OR 'mody':ti,ab OR 'ketosis resistant diabetes mellitus':ti,ab OR 'maturity onset diabetes mellitus':ti,ab OR 'non insulin dependent diabetes mellitus':ti,ab OR 'stable diabetes mellitus':ti,ab OR 'type ii diabetes mellitus':ti,ab OR 'slow onset diabetes mellitus':ti,ab OR 'type 2 diabetes mellitus':ti,ab OR 'type 2 diabetes':ti,ab OR 'adult onset diabetes mellitus':ti,ab

#3. 'mulberry twig alkaloids tablets':ti,ab OR 'mulberry twig alkaloids':ti,ab OR 'mulberry twig':ti,ab OR 'ramulus mori':ti,ab OR 'sangzhi':ti,ab

#2. 'non insulin dependent diabetes mellitus'/exp

#1. 'Mulberry Twig Alkaloids'/exp

Cochrane Library

#1 MeSH descriptor: [Mulberry Twig Alkaloids]

#2 MeSH descriptor: [Diabetes Mellitus, Type 2]

#3 (Mulberry Twig Alkaloids Tablets):ti,ab,kw OR (Mulberry Twig Alkaloids):ti,ab,kw OR (Mulberry Twig):ti,ab,kw OR (Ramulus Mori):ti,ab,kw OR (Sangzhi):ti,ab,kw

#4 (NIDDM):ti,ab,kw OR (MODY):ti,ab,kw OR (Ketosis Resistant Diabetes Mellitus):ti,ab,kw OR (Maturity Onset Diabetes Mellitus):ti,ab,kw OR (non-insulin-dependent diabetes mellitus):ti,ab,kw

#5 (Stable Diabetes Mellitus):ti,ab,kw OR (Type II Diabetes Mellitus):ti,ab,kw OR (Slow Onset Diabetes Mellitus):ti,ab,kw OR (Type 2 Diabetes):ti,ab,kw OR (Adult Onset Diabetes Mellitus):ti,ab,kw

#6 #1 OR #3

#7 #2 OR #4 OR #5

#8 #6 AND #7

Clinicaltrials.gov

Condition or disease: Diabetes Mellitus, Type 2

Intervention/treatment: Mulberry Twig Alkaloids

Study type: Intervention Studies (Clinical Trials)

Study Results: Study With Results

Ovid

1 (Mulberry Twig Alkaloids Tablets or Mulberry Twig Alkaloids or Mulberry Twig or Ramulus Mori or Sangzhi).m_titl.

2 (NIDDM or MODY or Ketosis Resistant Diabetes Mellitus or Maturity Onset Diabetes Mellitus or Non Insulin Dependent Diabetes Mellitus or Stable Diabetes Mellitus or Type II Diabetes Mellitus or Slow Onset Diabetes Mellitus or Type 2 Diabetes Mellitus or Type 2 Diabetes or Adult Onset Diabetes Mellitus).m_titl.

3 1 and 2

CINAHL

S1 SU Mulberry Twig Alkaloids Tablets OR SU Mulberry Twig Alkaloids OR SU Mulberry Twig OR SU Ramulus Mori OR SU Sangzhi

S2 SU Type 2 Diabetes Mellitus OR SU NIDDM OR SU MODY OR SU Ketosis Resistant Diabetes Mellitus OR SU Maturity Onset Diabetes Mellitus OR SU Non Insulin Dependent Diabetes Mellitus OR SU Stable Diabetes Mellitus OR SU Type 2 Diabetes OR SU Adult Onset Diabetes Mellitus OR SU Type II Diabetes Mellitus OR SU Slow Onset Diabetes Mellitus

S3 S1 AND S2

**
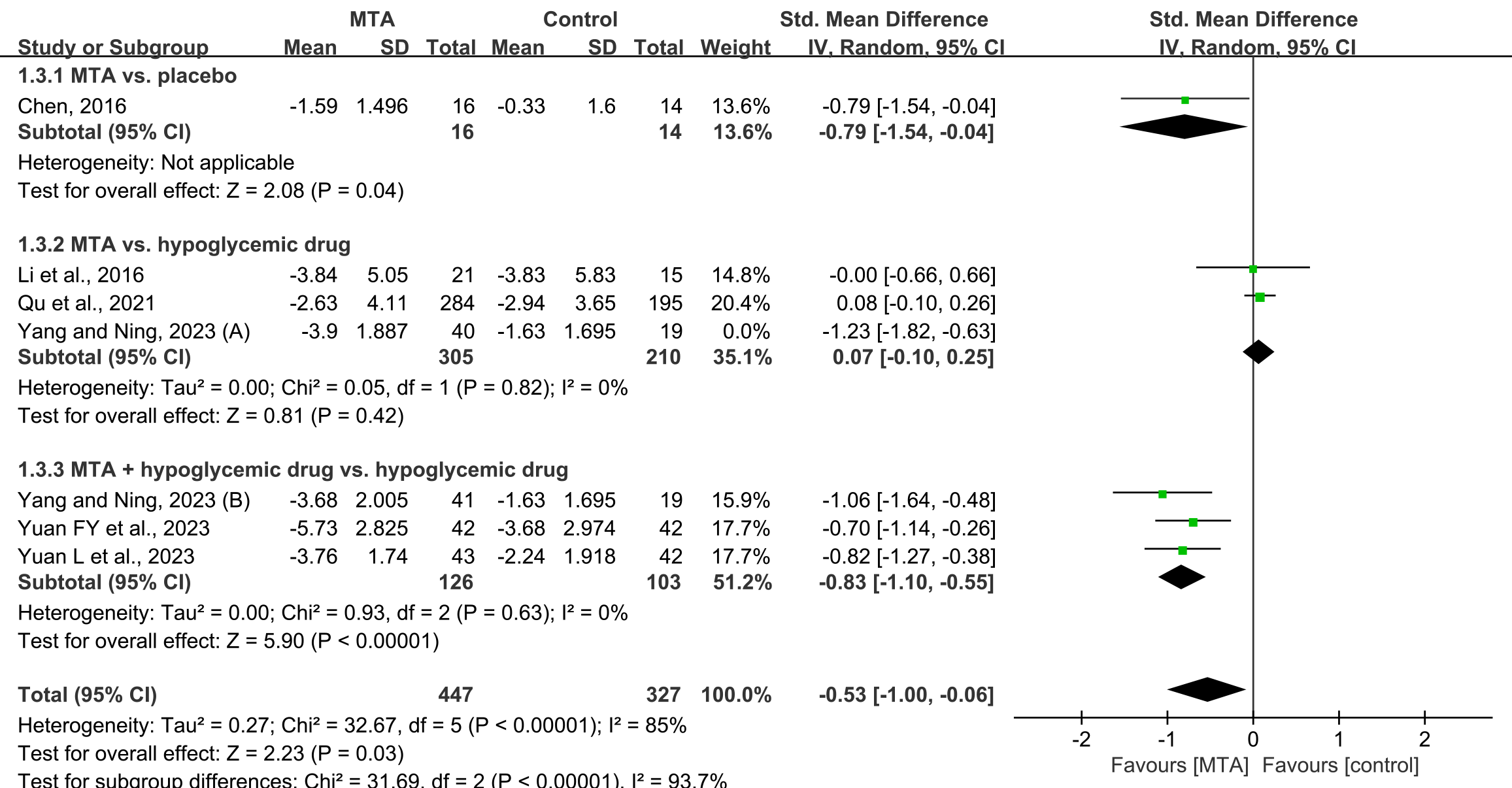
**

**Supplementary Figure S1.** Sensitivity analysis of 2hPG outcome after excluded 1 study (Yang & Ning, 2023).


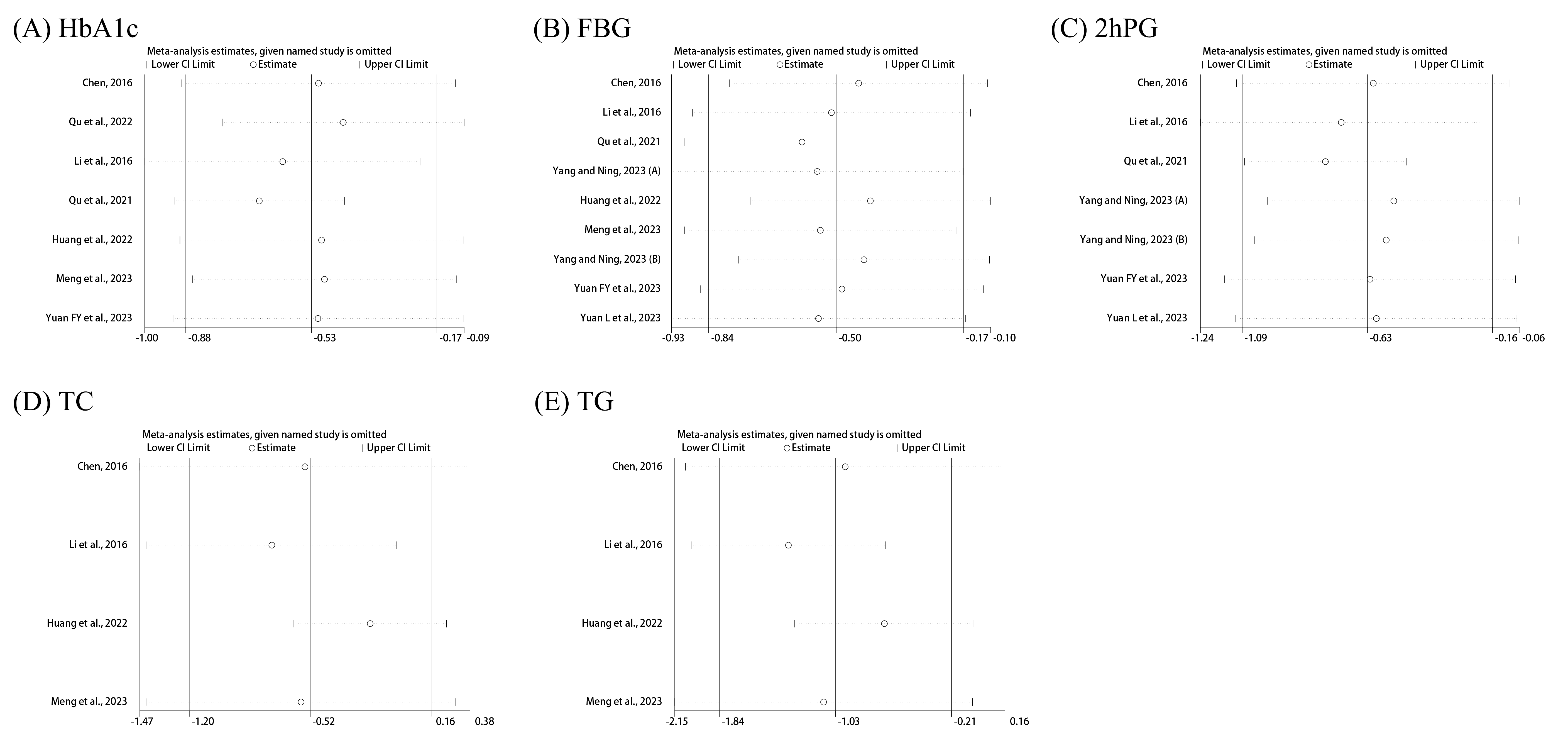


**Supplementary Figure S2.** Sensitivity analysis plots.


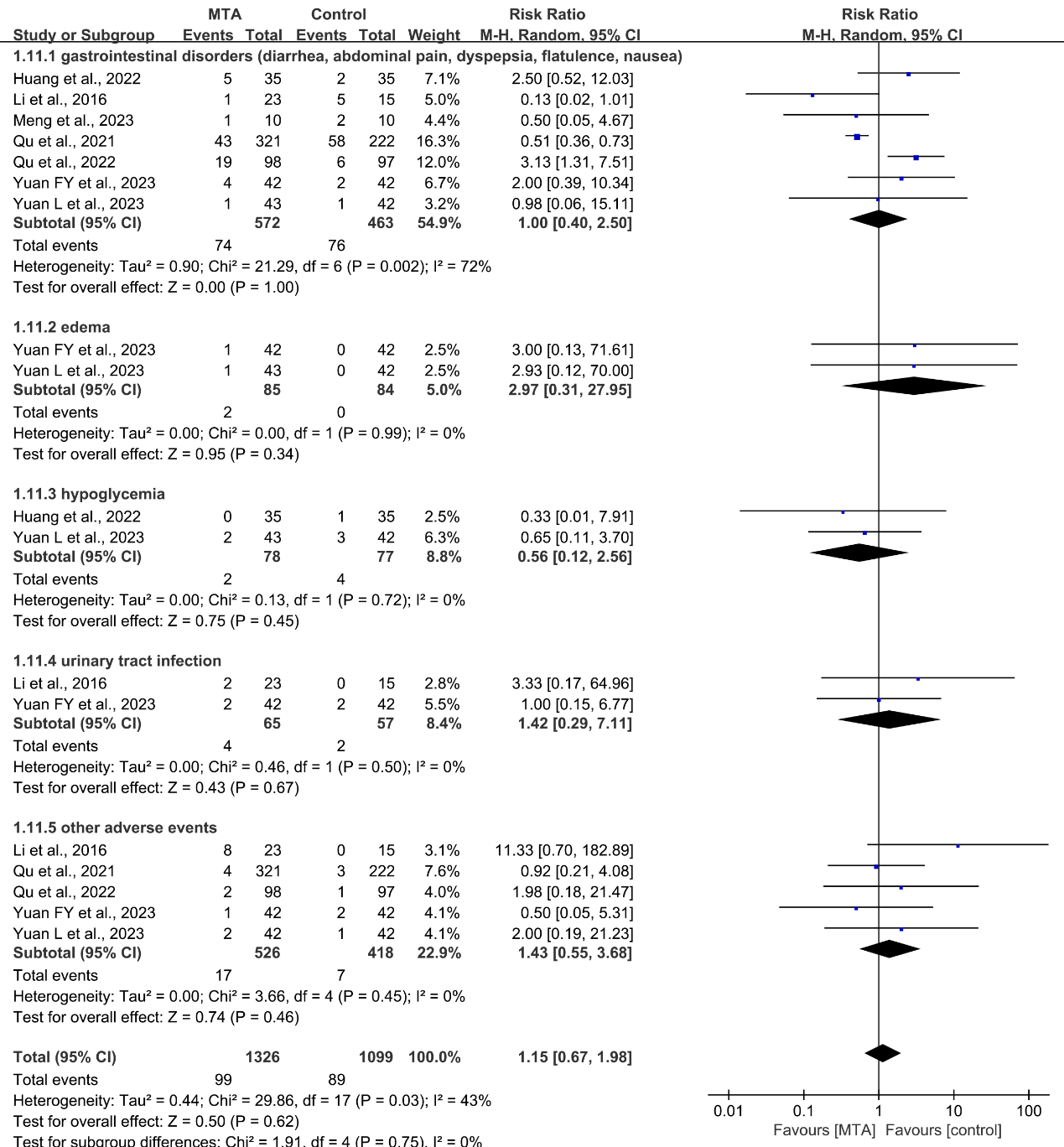


**Supplementary Figure S3.** Subgroup analysis of adverse events according to type.


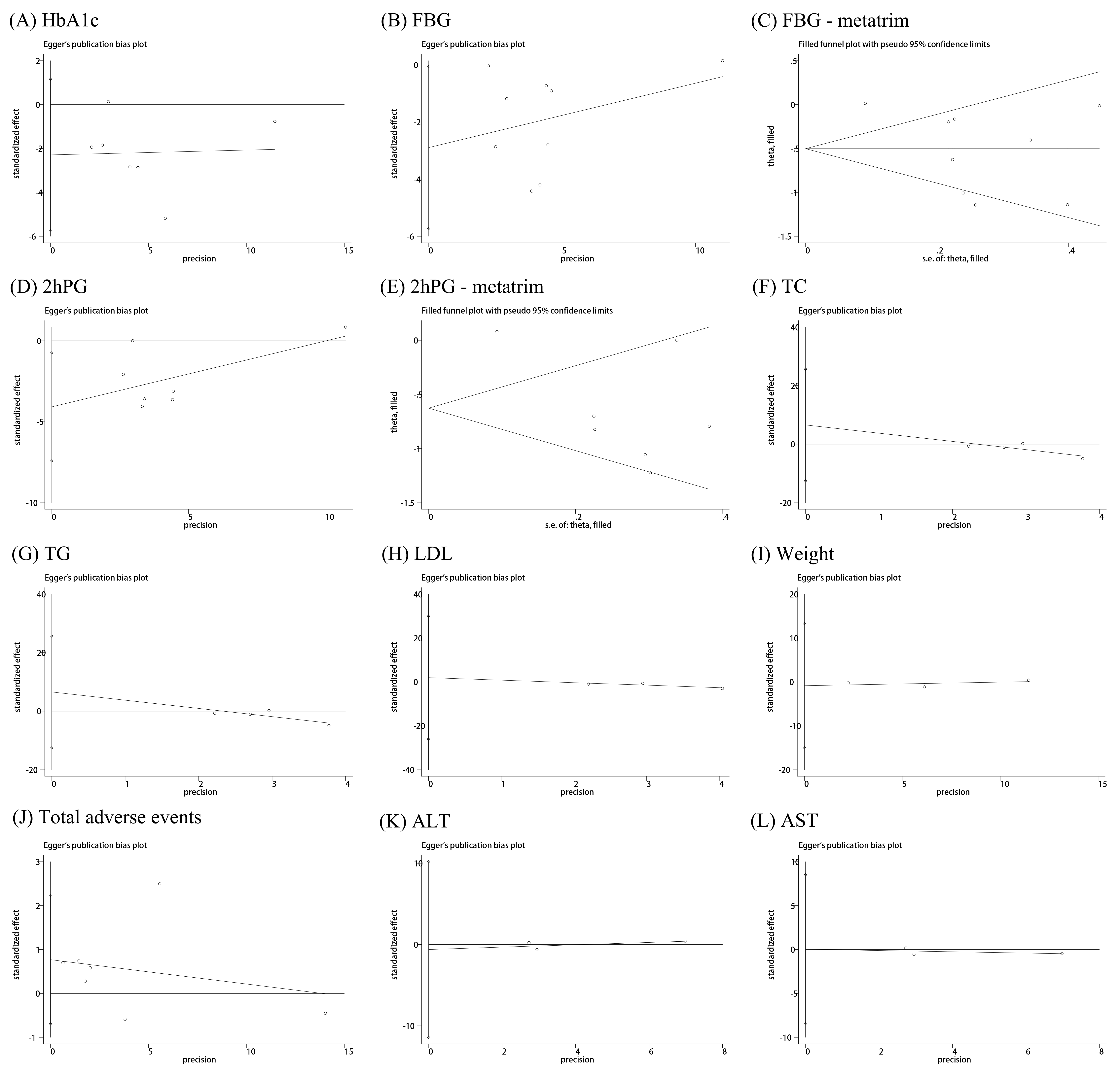


**Supplementary Figure S4**. Publication bias plots.

**Supplementary Table S2**. Egger test of outcomes.

| Outcome | Coef. | Std. Err. | t | *P*>\|t\| | [95% Conf. Interval] |
| --- | --- | --- | --- | --- | --- |
| HbA1c | -2.292178 | 1.341809 | -1.71 | 0.148 | [-5.741408 1.157052] |
| FBG | -2.893359 | 1.200788 | -2.41 | 0.047 | [-5.732772 -0.0539455] |
| 2hPG | -4.079502 | 1.297863 | -3.14 | 0.026 | [-7.415765 -0.7432388] |
| TC | 6.554765 | 4.434698 | 1.48 | 0.277 | [-12.5262 25.63573] |
| TG | 5.152998 | 7.024745 | 0.73 | 0.540 | [-25.07204 35.37804] |
| LDL | 1.944928 | 2.202534 | 0.88 | 0.539 | [-26.04092 29.93077] |
| Body weight | -0.8544408 | 1.1126 | -0.77 | 0.583 | [-14.99137 13.28249] |
| total adverse events | 0.7690615 | 0.5685477 | 1.35 | 0.234 | [-.6924368 2.23056] |
| ALT | -0.6140993 | 0.8505491 | -0.72 | 0.602 | [-11.42135 10.19315] |
| AST | 0.0348239 | 0.6669813 | 0.05 | 0.967 | [-8.439978 8.509625] |


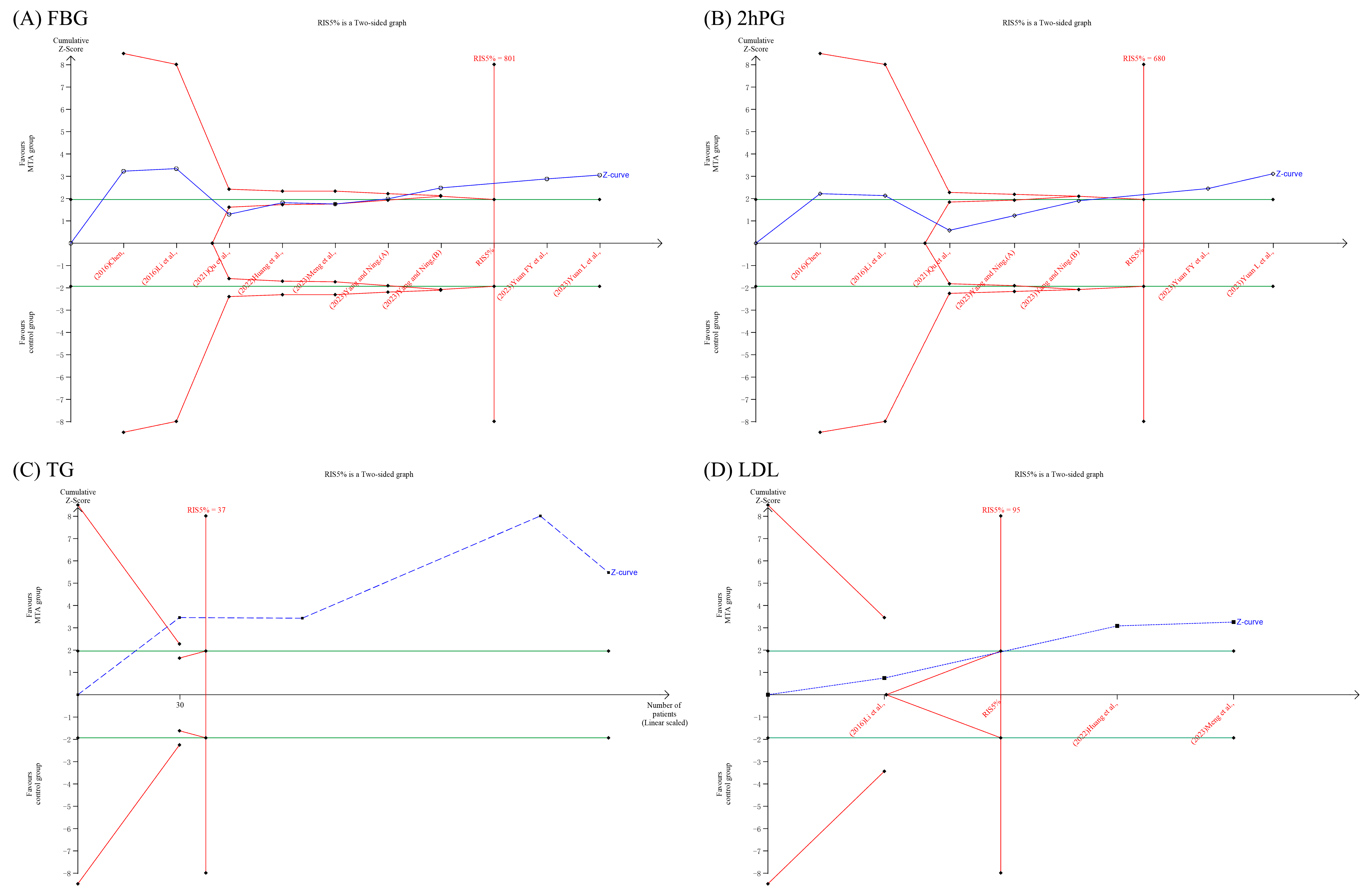


**Supplementary Figure S5**. Trial sequential analysis of FBG, 2hPG, TG, and LDL outcomes.

**Supplementary Table S3**. GRADE evaluation of evidence quality.

| **MTA for T2DM** | | | | | |
| --- | --- | --- | --- | --- | --- |
| **Outcomes** | **No of Participants (studies)** Follow up | **Quality of the evidence** (GRADE) | **Relative effect (95% CI)** | **Anticipated absolute effects** | |
|  |  |  |  |  | |
|  |  |  |  | **Risk with Control** | **Risk difference with T2DM** (95% CI) |
| **HbA1c** | 934 (7 studies) | ⊕⊕⊕⊝ **MODERATE** due to indirectness |  |  | The mean hba1c in the intervention groups was **0.53 standard deviations lower** (0.88 to 0.17 lower) |
| **FBG** | 986 (8 studies) | ⊕⊕⊝⊝ **VERY** **LOW** due to risk of bias, indirectness, publication bias |  |  | The mean fbg in the intervention groups was **0.50 standard deviations lower** (0.84 to 0.17 lower) |
| **2hPG** | 871 (6 studies) | ⊕⊝⊝⊝ **VERY LOW** due to risk of bias, indirectness, publication bias |  |  | The mean 2hpg in the intervention groups was **0.63 standard deviations lower** (1.09 to 0.16 lower) |
| **TC** | 156 (4 studies) | ⊕⊕⊝⊝ **LOW** due to indirectness, imprecision |  |  | The mean tc in the intervention groups was **0.52 standard deviations lower** (1.2 lower to 0.16 higher) |
| **TG** | 156 (4 studies) | ⊕⊕⊝⊝ **LOW** due to indirectness, imprecision |  |  | The mean tg in the intervention groups was **1.03 standard deviations lower** (1.84 to 0.21 lower) |
| **LDL** | 126 (3 studies) | ⊕⊕⊝⊝ **LOW** due to indirectness, imprecision |  |  | The mean tg in the intervention groups was 0.54 **standard deviations lower** (0.90 to 0.19 lower) |
| **HDL** | 56 (2 studies) | ⊕⊕⊝⊝ **VERY** **LOW** due to indirectness, imprecision |  |  | The mean tg in the intervention groups was 0.52 **standard deviations lower** (1.24 to 2.29 lower) |
| **total adverse events** | 1063 (8 studies) | ⊕⊕⊝⊝ **LOW** due to risk of bias, indirectness | **RR 1.08**  (0.95 to 1.22) | **Study population** | |
|  |  |  |  | **398 per 1000** | **32 more per 1000** (from 20 fewer to 88 more) |
|  |  |  |  | **Moderate** | |
|  |  |  |  | **131 per 1000** | **10 more per 1000** (from 7 fewer to 29 more) |
| **ALT** | 261 (3 studies) | ⊕⊕⊝⊝ **LOW** due to indirectness, imprecision |  |  | The mean alt in the intervention groups was **0.03 standard deviations higher** (0.22 lower to 0.27 higher) |
| **AST** | 261 (3 studies) | ⊕⊕⊝⊝ **LOW** due to indirectness, imprecision |  |  | The mean ast in the intervention groups was **0.07 standard deviations lower** (0.31 lower to 0.18 higher) |
| **Cr** | 225 (2 studies) | ⊕⊕⊝⊝ **LOW** due to indirectness, imprecision |  |  | The mean cr in the intervention groups was **0.08 standard deviations higher** (0.19 lower to 0.34 higher) |
| **Body weight** | 714 (3 studies) | ⊕⊕⊝⊝ **LOW** due to indirectness, imprecision |  |  | The mean weight in the intervention groups was **0.02 standard deviations lower** (0.17 lower to 0.13 higher) |
| **BMI** | 694 (2 studies) | ⊕⊕⊝⊝ **LOW** due to indirectness, imprecision |  |  | The mean tg in the intervention groups was 0**.03 standard deviations lower** (0.12 lower to 0.18 higher) |
| GRADE Working Group grades of evidence **High quality:** Further research is very unlikely to change our confidence in the estimate of effect.  **Moderate quality:** Further research is likely to have an important impact on our confidence in the estimate of effect and may change the estimate. **Low quality:** Further research is very likely to have an important impact on our confidence in the estimate of effect and is likely to change the estimate. **Very low quality:** We are very uncertain about the estimate. | | | | | |

**Supplementary Table S4**. PRISMA 2020 checklist.

| **Section and Topic** | **Item #** | **Checklist item** | **Location where item is reported** |
| --- | --- | --- | --- |
| **TITLE** | | |  |
| Title | 1 | Identify the report as a systematic review. | √ |
| **ABSTRACT** | | |  |
| Abstract | 2 | See the PRISMA 2020 for Abstracts checklist. | √ |
| **INTRODUCTION** | | |  |
| Rationale | 3 | Describe the rationale for the review in the context of existing knowledge. | √ |
| Objectives | 4 | Provide an explicit statement of the objective(s) or question(s) the review addresses. | √ |
| **METHODS** | | |  |
| Eligibility criteria | 5 | Specify the inclusion and exclusion criteria for the review and how studies were grouped for the syntheses. | √ |
| Information sources | 6 | Specify all databases, registers, websites, organisations, reference lists and other sources searched or consulted to identify studies. Specify the date when each source was last searched or consulted. | √ |
| Search strategy | 7 | Present the full search strategies for all databases, registers and websites, including any filters and limits used. | √ |
| Selection process | 8 | Specify the methods used to decide whether a study met the inclusion criteria of the review, including how many reviewers screened each record and each report retrieved, whether they worked independently, and if applicable, details of automation tools used in the process. | √ |
| Data collection process | 9 | Specify the methods used to collect data from reports, including how many reviewers collected data from each report, whether they worked independently, any processes for obtaining or confirming data from study investigators, and if applicable, details of automation tools used in the process. | √ |
| Data items | 10a | List and define all outcomes for which data were sought. Specify whether all results that were compatible with each outcome domain in each study were sought (e.g. for all measures, time points, analyses), and if not, the methods used to decide which results to collect. | √ |
|  | 10b | List and define all other variables for which data were sought (e.g. participant and intervention characteristics, funding sources). Describe any assumptions made about any missing or unclear information. | √ |
| Study risk of bias assessment | 11 | Specify the methods used to assess risk of bias in the included studies, including details of the tool(s) used, how many reviewers assessed each study and whether they worked independently, and if applicable, details of automation tools used in the process. | √ |
| Effect measures | 12 | Specify for each outcome the effect measure(s) (e.g. risk ratio, mean difference) used in the synthesis or presentation of results. | √ |
| Synthesis methods | 13a | Describe the processes used to decide which studies were eligible for each synthesis (e.g. tabulating the study intervention characteristics and comparing against the planned groups for each synthesis (item #5)). | √ |
|  | 13b | Describe any methods required to prepare the data for presentation or synthesis, such as handling of missing summary statistics, or data conversions. | √ |
|  | 13c | Describe any methods used to tabulate or visually display results of individual studies and syntheses. | √ |
|  | 13d | Describe any methods used to synthesize results and provide a rationale for the choice(s). If meta-analysis was performed, describe the model(s), method(s) to identify the presence and extent of statistical heterogeneity, and software package(s) used. | √ |
|  | 13e | Describe any methods used to explore possible causes of heterogeneity among study results (e.g. subgroup analysis, meta-regression). | √ |
|  | 13f | Describe any sensitivity analyses conducted to assess robustness of the synthesized results. | √ |
| Reporting bias assessment | 14 | Describe any methods used to assess risk of bias due to missing results in a synthesis (arising from reporting biases). | √ |
| Certainty assessment | 15 | Describe any methods used to assess certainty (or confidence) in the body of evidence for an outcome. | √ |
| **RESULTS** | | |  |
| Study selection | 16a | Describe the results of the search and selection process, from the number of records identified in the search to the number of studies included in the review, ideally using a flow diagram. | √ |
|  | 16b | Cite studies that might appear to meet the inclusion criteria, but which were excluded, and explain why they were excluded. | √ |
| Study characteristics | 17 | Cite each included study and present its characteristics. | √ |
| Risk of bias in studies | 18 | Present assessments of risk of bias for each included study. | √ |
| Results of individual studies | 19 | For all outcomes, present, for each study: (a) summary statistics for each group (where appropriate) and (b) an effect estimate and its precision (e.g. confidence/credible interval), ideally using structured tables or plots. | √ |
| Results of syntheses | 20a | For each synthesis, briefly summarise the characteristics and risk of bias among contributing studies. | √ |
|  | 20b | Present results of all statistical syntheses conducted. If meta-analysis was done, present for each the summary estimate and its precision (e.g. confidence/credible interval) and measures of statistical heterogeneity. If comparing groups, describe the direction of the effect. | √ |
|  | 20c | Present results of all investigations of possible causes of heterogeneity among study results. | √ |
|  | 20d | Present results of all sensitivity analyses conducted to assess the robustness of the synthesized results. | √ |
| Reporting biases | 21 | Present assessments of risk of bias due to missing results (arising from reporting biases) for each synthesis assessed. | √ |
| Certainty of evidence | 22 | Present assessments of certainty (or confidence) in the body of evidence for each outcome assessed. | √ |
| **DISCUSSION** | | |  |
| Discussion | 23a | Provide a general interpretation of the results in the context of other evidence. | √ |
|  | 23b | Discuss any limitations of the evidence included in the review. | √ |
|  | 23c | Discuss any limitations of the review processes used. | √ |
|  | 23d | Discuss implications of the results for practice, policy, and future research. | √ |
| **OTHER INFORMATION** | | |  |
| Registration and protocol | 24a | Provide registration information for the review, including register name and registration number, or state that the review was not registered. | √ |
|  | 24b | Indicate where the review protocol can be accessed, or state that a protocol was not prepared. | √ |
|  | 24c | Describe and explain any amendments to information provided at registration or in the protocol. | √ |
| Support | 25 | Describe sources of financial or non-financial support for the review, and the role of the funders or sponsors in the review. | √ |
| Competing interests | 26 | Declare any competing interests of review authors. | √ |
| Availability of data, code and other materials | 27 | Report which of the following are publicly available and where they can be found: template data collection forms; data extracted from included studies; data used for all analyses; analytic code; any other materials used in the review. | √ |
